# Bone Marrow Aplasia after CAR-T-Cell Therapy for Relapsed/Refractory Burkitt’s Lymphoma

**DOI:** 10.3390/medsci11040067

**Published:** 2023-10-12

**Authors:** Troy J. Kenkel, Nithya Sridhar, Lindsay R. Hammons, Maria Hintzke, Nirav N. Shah

**Affiliations:** 1 Department of General Internal Medicine, Medical College of Wisconsin, Milwaukee, WI 53226, USA; tkenkel@mcw.edu; 2 Department of Internal Medicine, Baylor College of Medicine, Houston, TX 77030, USA; nithya.sridhar@bcm.edu; 3 Division of Hematology and Oncology, Medical College of Wisconsin, Milwaukee, WI 53226, USA; lindsay.hammons.md@gmail.com; 4 Department of Pathology & Laboratory Medicine, Medical College of Wisconsin, Milwaukee, WI 53226, USA; mhintzke@mcw.edu

**Keywords:** chimeric antigen receptor T-cell, Burkitt lymphoma, aplastic anemia, immune effector cell-associated hemophagocytic lymphohistiocytosis-like syndrome

## Abstract

Chimeric antigen receptor T-cells (CAR-T) are now a standard approach for treating relapsed/refractory B-cell lymphomas. Immune effector cell-associated hemophagocytic lymphohistiocytosis-like syndrome (IEC-HS) is a newly described entity that can manifest following CAR-T. Bone marrow (BM) aplasia is an uncommon manifestation of IEC-HS reported after CAR-T-cell therapy and is defined as the reduction or absence of hematopoietic progenitor cells resulting in severe pancytopenia. We describe the case of a 44-year-old female with relapsed/refractory Burkitt lymphoma (BL) who received treatment with lisocabtagene maraleucel with her post-CAR-T course complicated by cytokine release syndrome (CRS) and IEC-HS ultimately leading to persistent BM aplasia. She underwent a rescue allogeneic stem cell transplant but ultimately succumbed to progressive disease. IEC-HS is an increasingly recognized complication that occurs after CAR-T treatments that can result in aplasia, a dangerous complication with serious sequelae including infection, transfusion dependence, and high risk for hemorrhage. The underlying mechanism is poorly understood, and further studies are needed to understand how to treat it better.

## 1. Background

In recent years, the development of chimeric antigen receptor T-cell (CAR-T) therapies has revolutionized the management of relapsed/refractory (R/R) B-cell malignancies [1]. CAR-T treatment is currently FDA-approved across multiple hematological malignancies, including B-cell acute lymphoblastic leukemia, diffuse large B-cell lymphoma and multiple myeloma. However, the role of these therapies in less common but aggressive histologies such as Burkitt lymphoma (BL) and Richter’s transformation is not as well defined [2].

BL is a rare and very aggressive form of B-cell non-Hodgkin’s lymphoma (NHL), hallmarked by aberrations in *MYC* [3]. BL has three distinct subclasses: endemic, sporadic, and those immunodeficiency-associated [4]. Due to its highly aggressive nature, BL is typically very responsive to intensive chemotherapy. However, when treatment fails, relapsed BL has an especially poor prognosis [3]. Treatment options are limited but include second-line high-dose chemotherapy with autologous stem cell transplantation or allogeneic transplantation. As the disease is rare and uncommonly relapses, CAR-T treatment for this population has been difficult to study [5]. Limited case reports and case series have described mixed outcomes in BL. Durable complete remission following CAR-T has been shown in some cases of BL, whereas the rapid progression of disease or complications of the treatment have led to death in other patients (see Table 1) [6,7,8,9,10,11,12,13,14].

Although not common, there are a few case reports of bone marrow (BM) failure after CAR-T in aggressive NHL (see Table 2) [15,16,17,18,19], and our case describes the first instance of post-treatment bone marrow aplasia in an adult with BL. Bone marrow aplasia is characterized by hematopoietic failure in the BM, leading to a decrease or absence of hematopoietic progenitors [20]. Although the mechanism of aplasia is not clearly defined, it is likely either caused by an intrinsic dysfunction of the bone marrow or an extrinsic suppression of hematopoietic stem cells, usually caused by infections, congenital chromosome diseases, drugs, and autoimmune conditions, among others [20]. One theory postulates an abnormal T-cell response in which dysfunction of T-helper cells leads to cytopenias in the peripheral blood, providing a potential mechanistic explanation of CAR-T-mediated aplasia [20]. HLH-like toxicities termed immune effector cell hemophagocytic lymphohistiocytosis-like syndrome (IEC-HS) [21] is a recently described phenomenon and may play a role in prolonged cytotoxicity as well. It refers to a hyperinflammatory syndrome that is seen following immune effector cell therapy such as CAR-T. Clinical features include hyperferritinemia, cytopenias, and coagulopathy and may provide a pathologic basis for aplasia following CAR-T. Here, we report a novel case of aplasia in a patient with R/R BL after receiving CAR-T.

## 2. Case Description

**Figure 1:**
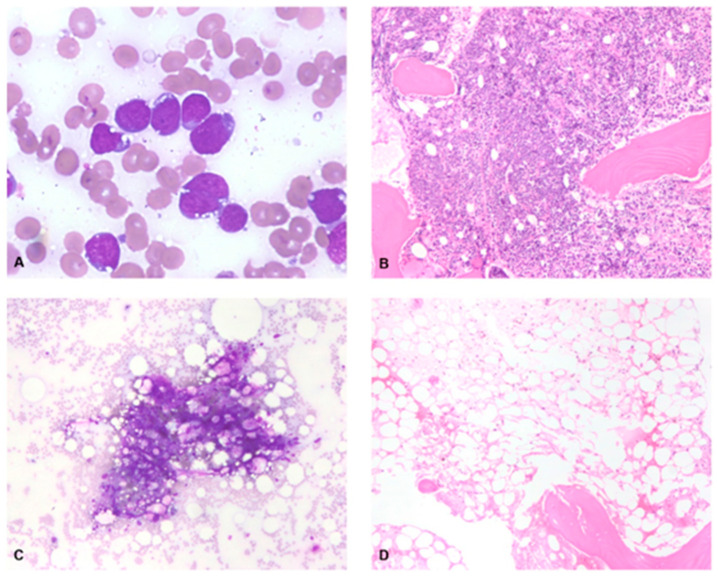
BM pathology post-CAR-T day +48. (**A**) Bone marrow aspirate, modified Wright-Giemsa, 50X, pre-CAR-T, shows numerous large lymphoma cells. (**B**) Bone marrow core biopsy, H&E, 20X, pre-CAR-T, shows a hypercellular bone marrow with an interstitial and nodular infiltrate of large lymphoma cells. (**C**) Bone marrow aspirate, modified Wright-Giemsa, 20X, post-CAR-T, shows essentially acellular spicules. (**D**) Bone marrow core biopsy, H&E, 20X, post-CAR-T, shows aplasia.

The patient is a 43-year-old female with R/R stage IV BL with cerebral spinal fluid (CSF), bone marrow, and extranodal involvement when diagnosed. Her initial treatment included the modified McGrath regimen with rituximab [22]. Interim evaluation after two cycles demonstrated complete remission (CR) by PET (Deauville 1), bone marrow biopsy (*MYC* FISH negative), and CSF. Unfortunately, she progressed after three months with marrow, leptomeningeal, and CSF involvement. She then received the MATRIX regimen [23] along with intrathecal methotrexate and achieved CR. She subsequently underwent stem cell collection with a plan for autologous hematopoietic stem cell transplant (auto-HCT) consolidation. Pre-transplant, she developed new diplopia, and imaging demonstrated isolated CNS relapse. She was also neutropenic at this time and required five days of granulocyte colony-stimulating factor (G-CSF) for count recovery. She received emergent craniospinal irradiation with dexamethasone and proceeded immediately to a rituximab/thiotepa/busulfan/cyclophosphamide conditioned auto-HCT. Despite high-dose chemotherapy, her day +30 marrow demonstrated relapsed BL—she was given rituximab–polatuzumab and underwent apheresis for anti-CD19 CAR-T therapy (lisocabtagene maraleucel). Pre-CAR-T, her BL rapidly progressed with widespread systemic (bone marrow with 90% involvement; see Figure 1) and CSF involvement. She was also found to be COVID-19 + at this time, but given the emergent clinical need, she proceeded with lymphodepletion and CAR-T infusion. She underwent pretreatment with fludarabine and cyclophosphamide prior to infusion with lisocabtagene maraleucel.

Her post-CAR-T treatment course was complicated by Grade 1 CRS (Tmax of 102.7) managed with tocilizumab. Following the resolution of CRS, she developed pancytopenia and HLH-like toxicities consistent with the newly defined entity IEC-HS [21] manifesting with hyperferritinemia (peak ferritin ≥ 100,000 ng/mL), transaminitis, hyperbilirubinemia (peak Tbili 7.9 mg/dL), and hypofibrinogenemia (nadir = 100 mg/dL). She was treated with dexamethasone and anakinra, with rapid improvement in symptoms and biochemical markers.

**Figure 2:**
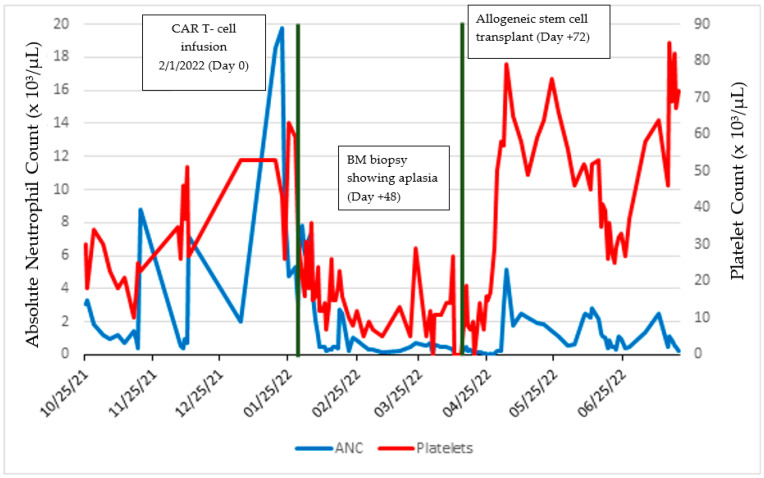
Timeline of absolute neutrophil count and platelet levels throughout patient’s course.

Her initial response to CAR-T cell therapy was consistent with a CR confirmed by PET/CT, bone marrow biopsy, CSF analysis, and MRI brain. Unfortunately, she developed recurrent pancytopenia. Bone marrow performed on post-CAR-T day +48 revealed < 5% cellularity with only the presence of small mature lymphocytes, stromal cells, and histiocytes concerning aplasia (see Figure 1). Despite having received a growth factor, she had no improvement in her counts. As a previous donor evaluation had confirmed a matched sibling, she proceeded with rescue allogeneic hematopoietic stem cell transplant (allo-HCT) from a matched related donor on day +65 (see Figure 2).

On day +17, after her allo-HCT, she had neutrophil engraftment. Initial chimerisms were 100% and 98% for CD33 and CD3, respectively. Unfortunately, on post-allogeneic transplant day +55, she developed new-onset ascites and was found to have relapsed BL. She received blinatumomab with no clinical response and ultimately entered home hospice and died of progressive lymphoma.3. Discussion and Conclusions

The number of patients receiving CAR-T treatment for R/R B-cell malignancies is increasing with more approvals in different subtypes of lymphoma. While this treatment can lead to durable remissions in patients with refractory lymphoma, both short-term and long-term toxicities can impact patient outcomes. We report a patient with BL who, post-CD19 CAR-T cell therapy, experienced both CRS and IEC-HS, resulting in pancytopenia and BM findings most consistent with aplasia requiring emergent transplant for hematopoietic recovery. Hematologic toxicities, especially aplasia, defined as the reduction or absence of hematopoietic progenitor cells, can be detrimental and are seldom described in the literature [10]. Although prolonged cytopenias can occur after a patient’s post-CAR-T cell therapy, persistent pancytopenia unresponsive to growth factors is a concerning finding [17]. In the ZUMA-7 trial, 91% of patients who received second-line axicabtagene ciloleucel for the treatment of large B-cell lymphoma developed grade 3 or greater cytopenias and 29% developed prolonged cytopenias [24], but frank aplasia was not reported. In the TRANSCEND trial using lisocabtagene maraleucel as second-line therapy in R/R large B cell lymphoma, 48% of patients had neutropenia, 21% had leukopenia, and 20% had thrombocytopenia, while 37% developed prolonged cytopenias [11]. While cytopenias following CAR-T are a commonly reported adverse event, the severity and duration can be quite variable. Severe cases leading to aplasia are much more rare, with unclear prevalence at this time. The association with hyperinflammatory clinical features is also being seen more. IEC-HS is becoming increasingly recognized and may be an underlying etiology related to the development of aplasia.

IEC-HS is defined as a hyperinflammatory syndrome that is hallmarked by features of macrophage activation/HLH, immune effector cell therapy as the primary etiology, and is associated with cytopenias, hyperferritinemia and coagulopathy [21]. CRS is a commonly seen entity following CAR-T therapy with an overlap in clinical presentation. However, effects from IEC-HS are often more delayed, and diagnosis is dependent on differentiation from CRS. Frequently, symptoms of IEC-HS will be seen following the resolution of CRS. Importantly, IEC-HS is not seen concurrently with CRS. The only laboratory requirement for the diagnosis of IEC-HS is hyperferritinemia, which does not have strict parameters, instead using “rapidly increasing” to allow for physician discretion. Grading is on a scale of 1–5, ranging from asymptomatic to increasing severity, with stage 5 representing death. IEC-HS is a newly described condition, and as such, there are limited data guiding treatment, and it is based primarily on expert opinion. Presently, steroids +/− anakinra are recommended with ruxolitinib as third-line therapy for life-threatening toxicity. Treatment with these agents is not without adverse effects, including susceptibility to infection. IEC-HS requires further study, specifically its relation to severe cytopenias and BM aplasia, to better understand how to manage them.

Aplasia following CAR-T is a dangerous complication given an increased risk of infection, transfusion dependence, and increased risk of hemorrhage and major bleeding. Furthermore, rescue treatment with emergent allogeneic stem cell transplant is an option but remains a high-risk procedure difficult to implement in a patient following CAR-T cell therapy. Although the review of the literature comments on this finding in patients with a variety of hematological malignancies post-CAR-T cell therapy (see Table 2), our case describes the first instance of aplasia after CAR-T in a patient with BL.

The mechanism of CAR-T leading to aplasia is one that is unclearly defined. One possibility is cytokines interfering with hematological progenitor cell development, resulting in an aplastic crisis [15]. An alternate theory suggests that autoreactive T-cells incorrectly recognize hematopoietic progenitors in the BM and destroy them, resulting in aplasia [18]. The diagnosis of aplasia requires excluding other diagnoses, such as myelodysplastic syndrome or malignant infiltration of the bone marrow. The optimal treatment of CAR-T-associated aplasia is unclear, given the rarity of this complication. The approach used in this case of rescue allogeneic transplant was effective for hematopoietic recovery. However, allo-HCT has its own risks and complications. Other groups have utilized agents with known efficacy in aplasia, such as thrombopoietin receptor (TPO) agonists. In one case series of 6 patients, all experienced improvement in pancytopenia with a TPO agonist and with 5 having complete resolution [19]. In our case, the patient underwent an emergent allogeneic stem cell transplant due to persistent hypocellularity after her CAR-T. Although she tolerated the transplant well, her BL ultimately relapsed, and she opted for comfort care, with death following shortly after.

Overall, CAR-T is a new frontier in cancer therapy that is still being further developed. It has shown promising results in many hematologic malignancies [2]. But this success is not ubiquitous. CAR-T in BL has not been studied robustly to date, and the existing data are varied. Some studies have demonstrated complete response following CAR-T in BL, whereas others have shown rapid progression of disease with high mortality. In the cases exhibited in Table 1 [6,7,8,9,10,11,12,13,14], 76% of patients with R/R BL experienced CR following CAR-T. Although CR was achieved in the majority of these patients, the duration of response was widely variable—some experienced relapse and rapid progression while others achieved durable remission of many months. Sequential therapy with multiple CAR-T products directed at different antigens has been used as well [9,13]. While this strategy sometimes led to an improvement in the overall CR rate, whether this is due to the additive effect of CAR-T products or increased efficacy from alternative treatments is not clear and did not necessarily lead to better long-term outcomes. Aside from tumor response, these studies demonstrated that CAR-T in BL comes with a high degree of CRS burden, which carries significant morbidity and mortality. A total of 90% of patients experienced any grade CRS, while 26% experienced Grade III or higher. In addition, cytopenias are common post-CAR-T, although typically recovering quickly, making patients susceptible to severe infection. More recent data paint an even more dismal picture of outcomes in this group [25]. Of 13 patients that received CAR-T with R/R BL in this study, CR was seen in 53.6%, with only 30.8% having a sustained response of more than 6 months [25]. Median progression-free survival was 4.2 months [25]. Additional investigation is warranted to decipher more effective treatments in this patient population. What ultimately determines a patient’s response to CAR-T is not fully understood, and further work is still needed in this area.

Given the limited data for CAR-T in BL, an extremely aggressive malignancy with rapid proliferation, the overall outcomes and toxicity profile remain unclear. Some studies have shown durable response, while others show rapid progression with high mortality. Inflammatory side effects such as CRS and IEC-HS are frequently seen, as well as cytopenias. Our patient experienced severe CAR-T-associated toxicities consistent with IEC-HS, including hyperferritinemia and profound, prolonged cytopenias; subsequent aplasia that did not improve with standard growth factor support. The optimal treatment for this rare and unfortunate side effect has not been determined. Further studies of CAR-T and the mechanism of cellular toxicity, and the contribution of IEC-HS are needed to better understand this condition and how to best treat it.

## Figures and Tables

**Table 1:** Studies of the use of CAR-T therapy in Burkitt lymphoma.

Authors.	Age	Line of Treatment	Type of CAR-T	CRS	Best Response (# of Patients)	Survival (# of Patients)
Avigdor et al. (2018)	32	4	Anti-CD19	Grade II	CR	Deceased (1) 2 mo
Cheng et al. (2022)	47	4	Sequential allogeneic anti-CD22 and anti-CD19	Grade I	PR	Deceased (1) 1 mo
Du, Zhang (2020)	8	2	Sequential anti-CD19, anti-CD22, and anti-CD20	Grade III	CR	Alive (1) 16+ mo
Liu et al. (2019))	2.5–12 (23 patients)	Not reported	Anti-CD19, anti-CD22, anti-CD20	Grade I (6), Grade II (3), Grade III (8)	*1 CAR-T infusion*: CR (15), PR/NR (8) *2 CAR-T infusion:* CR (8), PR/NR (5) *3 CAR-T infusion:* CR (2), PR/NR(1)	Alive (19) 12+–36+ mo Deceased (4) 4–12 mo
DeceSeitter et al. (2022)	33, 45	3	Anti-CD19	Grade II (2)	CR (2)	Alive (2) 53+ mo and 58+ mo
Wang et al. (2021)	2	4	Anti-CD19	Grade II	CR	Alive (1) 16+ mo
Wu et al. (2022)	17–70 (28 patients)	3 (range 2–7)	Sequential anti-CD22 and anti-CD19	Grade I (16), Grade II (5), Grade III-IV (6)	CR (16), PR (3), NR (9)	Alive (16) 0.1+–58.6+mo Deceased (12) 0.1–5.3
Zhang et al. (2020)	6–10 (5 patients)	7	Anti-CD19 (5), anti-CD22 (2), anti-CD20 (1)	Grade I (3), Grade III (2)	*1 CAR-T infusion*: CR (3), PR/NR (2) *2 CAR-T infusion:* CR (1), PR/NR(1) *3 CAR-T infusion:* CR (1)	Alive (5) 12.4+–35.7+ mo
Zhou et al. (2021)	21–34 (6 patients)	3.5 (range 1–5)	Sequential anti-CD22 and anti-CD19	Grade I (5), Grade III (1)	CR (1), PR (2), NR (3)	Deceased (3) 1–10 mo Alive (3) 4+–37+ mo

CR—complete response. PR—partial response. NR—No response. +: alive at the end of the study period.

**Table 2:** Reports of hematologic toxicity following treatment with CAR-T therapy.

Authors	Lymphoma	CAR-T	CRS (Grade)	ICANS (Grade)	Hematotoxicity	Cytopenia Treatment
Rejeski, Wu (2022)	Richter-transformation DLBCL	Tisagenlecleucel	3	NR	Aplastic anemia	G-CSF, eltrombopag
Rejeski, Kunz (2021)	DLBCL	Axicabtagene ciloleucel	1	4	BM aplasia	Stem cell boost of autologous CD34+ cells
Baur (2021)	DLBCL	Axicabtagene ciloleucel	2	NR	Thrombocytopenia; aplasia	Romiplostim
Qasrawi (2020)	DLBCL	Axicabtagene ciloleucel	2	3	BM aplasia	Filgrastim, eltrombopag, allo-HCT
Beyar-Katz (2022)	DLBCL	Tisagenlecleucel, axicabtagene ciloleucel	NR	NR	BM aplasia	Eltrombopag, romiplostim
Current report (2023)	Burkitt’s lymphoma	Lisocabtagene maraleucel	1	NR	BM aplasia	Filgrastim, allo-HCT

NR—not reported. Definitions: diffuse large B-cell lymphoma (DLBCL), allogeneic hematopoietic stem cell transplant (allo-HCT), granulocyte colony-stimulating factor (G-CSF).

## Data Availability

The datasets used and/or analyzed during the current study are available from the corresponding author upon reasonable request.

## Abbreviations

R/R	Relapsed/refractory
BL	Burkitt lymphoma
CAR-T	Chimeric antigen receptor T-cell
CRS	Cytokine release syndrome
NHL	Non-Hodgkin’s lymphoma
ConCSF	Cerebrospinal fluid
CR	Complete remission
Auto-HCT	Autologous hematopoietic stem cell transplant
HLH	Hemophagocytic lymphohistiocytosis
Allo-HCT	Allogeneic hematopoietic stem cell transplant
IEC-HS	Immune effector cell-associated hemophagocytic lymphohistiocytosis-like syndrome
TPO	Thrombopoietin receptor
